# Severe Ketamine-Induced Uropathy Associated With Cholangiopathy Requiring Percutaneous Nephrostomy

**DOI:** 10.7759/cureus.30443

**Published:** 2022-10-18

**Authors:** Ngoda Manongi, Sean Ho Yoon, Jean J Luo, Priyank Trivedi

**Affiliations:** 1 Internal Medicine, New York Presbyterian Queens, Flushing, USA; 2 Pathology, New York Presbyterian Queens, Flushing, USA; 3 Critical Care Medicine, New York Presbyterian Queens, Flushing, USA

**Keywords:** bladder inflammation, hydronephrosis, bilateral nephrostomy tube, ketamine, ketamine-induced uropathy

## Abstract

Ketamine-induced uropathy (KIU) is becoming more prevalent as ketamine abuse becomes widespread. Recently, a plethora of reports have highlighted the association between recreational ketamine abuse and its deleterious effects including uropathy and cholangiopathy. However, there are hardly any reports that demonstrate the management of severe KIU in young people. We report a case of an Asian female in her late 20's with a 10-year history of ketamine abuse who presented to the emergency department with sharp, epigastric pain for two weeks. An evaluation revealed acute kidney injury and transaminitis with bilirubinemia. CT scan showed bladder and ureteral wall thickening with hydroureteronephrosis. The patient underwent percutaneous bilateral nephrostomy tubes placement, improving urine output and with acute kidney injury resolution. Repeat renal/bladder US showed near complete resolution of bilateral hydronephrosis. This case documents one of the first few documented cases of severe ketamine-induced uropathy with cholangiopathy necessitating bilateral nephrostomies in the continental U.S.

## Introduction

Ketamine-induced uropathy (KIU) is becoming more prevalent as ketamine abuse becomes widespread. Chronic recreational ketamine use can cause a syndrome of cystitis and contracted bladder [1]. In severe cases, permanent kidney damage can occur making patients dialysis-dependent. Moreover, ketamine has been shown to cause liver and biliary injury [2]. The mechanism of both ketamine-induced uropathy and cholangiopathy remains unknown. Lower urinary tract symptoms (LUTS) including hesitancy, poor stream, frequency, incontinence, and nocturia are the principal presenting symptoms in patients with ketamine abuse; however, ureteral inflammation, stricture, hydronephrosis, and impaired renal function can occur [3]. We report a case of a 29-year-old Asian female with a 10-year history of ketamine abuse who presented with abdominal pain, nausea, and vomiting and was found to have acute-on-chronic kidney disease with radiographic evidence of worsening, non-obstructive, severe bilateral hydronephrosis that required bilateral nephrostomies tubes in the acute phase of management. 

## Case presentation

**Figure 1:**
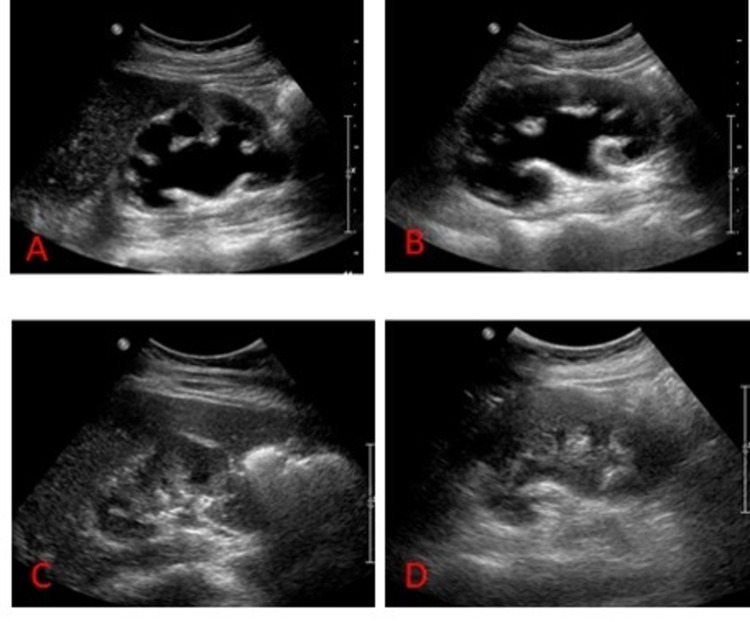
Severe bilateral hydronephrosis noted (right (A) and left (B) kidneys, respectively), likely slightly increased on the left. Renal atrophy/scar, and probable medical renal disease. Near complete resolution of bilateral hydronephrosis (right (C)and left (D) kidneys, respectively) 7 days status post bilateral nephrostomy placement.

**Figure 2:**
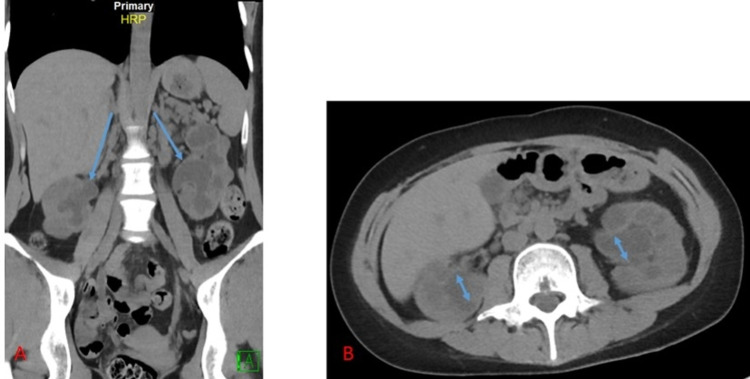
Non-contrast CT abdomen pelvis demonstrating ureteric wall thickening with hydroureteronephrosis (blue arrows), without nephrolithiasis.

**Figure 3:**
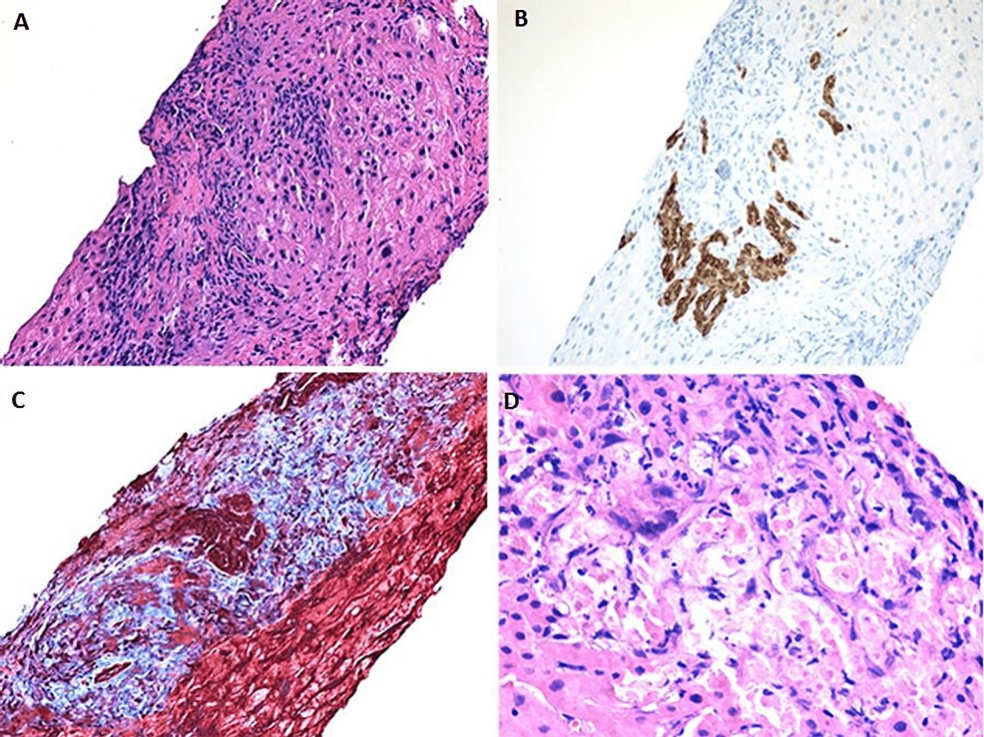
(A) Needle liver biopsy showing expanded portal tract with mixed lymphocytic and neutrophilic inflammation, ductular reaction and mild fibrosis; In the per-portal area, cholestasis found in hepatocytes and bile canaliculi (hematoxylin and eosin stain: x200). (B) Proliferative bile ductules (immunostain of CK7: x200). (C) Mild portal fibrosis (Mallory trichrome special stain: x200). (D) Liver injury with ballooning hepatocytes and neutrophilic infiltration (hematoxylin and eosin stain: x400).

A 29-year-old Asian female without significant medical history presented to the emergency department with sharp, epigastric pain for two weeks. The pain was associated with nausea and generalized weakness. Upon further questioning, the patient admitted to recreational ketamine use for 10 years. The abdominal exam was remarkable for epigastric tenderness. Initial blood work revealed mild leukocytosis without bands, hyponatremia, transaminitis, elevated creatinine, and positive urinalysis (Table 1). Urine output monitoring demonstrated oliguria. Renal ultrasound revealed severe bilateral hydronephrosis and scarring (Figures 1A, 1B). A computed tomography scan of the abdomen demonstrated bladder and ureteral wall thickening with hydroureteronephrosis (Figure 2). Magnet resonance cholangiopancreatography and endoscopic ultrasound were unremarkable. A liver biopsy showed bile ductule proliferation with associated neutrophilic infiltrate consistent with ketamine-induced changes and rare focal hepatocytic ballooning within the hepatic lobule (Figure 3). The Hepatitis panel revealed a positive hepatitis B virus viral load (HBV VL). Immunostains of liver biopsy for hepatitis B surface antigen (HBsAg) or hepatitis B core antigen (HBcAg) were negative. The patient underwent percutaneous bilateral nephrostomy tubes placement, improving urine output and resolution acute kidney injury resolution. Repeat renal/bladder US showed near complete resolution of bilateral hydronephrosis (Figures 1C, 1D). Transaminitis and hyperbilirubinemia markedly improved without intervention. The patient was discharged home on the 17th day after admission.

**Table 1:** Laboratory analysis

Laboratory profile at admission		Reference ranges
Hematology		
White blood cells (K/uL)	12.99	4.80 – 10.80
Differential count		
Neutrophils (%)	86.9	37 – 80
Lymphocytes (%)	5.7	15 – 40
Haemoglobin (g/dL)	12.1	11.7–15.3
Platelet count (K/uL)	314	150 – 400
Chemistry		
Sodium (mmol/L)	112	136 – 145
Potassium (mmol/L)	3.8	3.5 – 5.1
Creatinine (mg/dL)	6.98	0.70 –1.30
eGFR (mL/min/1.73 m^2^)	7	> = 60
Carbon dioxide (mmol/L)	12	22 – 29
Anion Gap	24	5 –17
Phosphorous (mg/dL)	2.1	0.5 – 1.6
Osmolality (mOsm/kg)	274	280 – 305
pH Venous	7.399	7.350 – 7.450
Alanine aminotransferase (units/L)	169	5 – 41
Aspartate aminotransferase (units/L)	224	5 – 40
Bilirubin, total (mg/dL)	3.7	0.0 – 1.2
Bilirubin, direct (mg/dL)	3.3	0.0 – 0.3
Alkaline phosphatase (units/L)	1737	40 – 130
Urinalysis		
Appearance	Clear	Clear
Color	Yellow	Yellow
Specific gravity	1.012	1.010 – 1.030
Bacteria	Negative	Negative
Squamous epith cells	5	0 – 5
Red blood cells (/HPF)	44	0 – 2
White blood cells (/HPF)	>100	0 – 3
Hyaline casts (/LPF)	>18	0 – 7
pH	7.5	5 – 8
Blood	Moderate	Negative
Ketones	Trace	Negative
Nitrites	Negative	Negative
Leukocyte esterase	Moderate	Negative

Unfortunately, the patient was readmitted to the hospital three months later due to a leaking left nephrostomy tube and positive urinalysis. In addition, her urine culture grew Streptococcus epididimus, Enterococcus faecium, and Aeromonas salmonicida for which the patient was started on antibiotics. The nephrostomy tubes were replaced with bilateral indwelling ureteral stents. The patient's condition improved and was discharged on the 10th day after re-admission. The patient had bilateral stents exchange at six months and one-year without acute complaints. 

## Discussion

Ketamine-induced uropathy (KIU) is becoming more prevalent in the U.S. Ketamine, which first became available in the mid-1960s, is a cyclohexanone derivative of phencyclidine (PCP). It is an NMDA receptor antagonist that blocks glutamate. Ketamine uses includes general anesthesia, procedural sedation, therapy-resistant depression, chronic pain, and its amnestic properties during surgical procedures. Ketamine is metabolized in the liver to its active metabolite norketamine which is then excreted in the urine [4]. A plethora of side effects have been reported including dizziness, arrhythmias, decreased respiratory drive, behavioral changes, amnesia, and hallucinations. Recreational ketamine abuse is well documented in East Asia because of its hallucinogenic effects and its use in the rave scene. However, chronic recreational ketamine use can cause structural damage to the renal and hepatobiliary systems [4,5]. Although the mechanism for hepatobiliary and urinary tract destruction in ketamine abuse remains unknown, severe cases can cause ureteral and biliary inflammation and strictures, hydronephrosis, and impaired renal and hepatic function. Studies have shown that ketamine and its metabolites directly impact urothelial cells and hepatocytes, causing microvascular changes that induce autoimmune responses [2-5]. As most patients are young, it is imperative that their upper urinary tract is protected to prevent chronic renal disease with a percutaneous nephrostomy or ureteral stenting in cases of severe hydronephrosis [6]. Percutaneous nephrostomy involves placing a plastic tube through the skin into the renal pelvis to drain urine trapped in the obstructed system. Consequently, the drainage and diversion of urine away from the bladder through percutaneous nephrostomy reduce lower urinary tract symptoms. Currently, only abstinence has been shown to reverse some of this ketamine's damage.

## Conclusions

The rise in widespread abuse of recreational ketamine in the U.S. is associated with new emerging renal and hepatobiliary diseases in our already stressed healthcare system. Our case emphasizes that chronic recreational ketamine use can cause structural damage to the kidneys, bladder, and hepatobiliary system. Although the mechanism for urinary tract and hepatobiliary destruction remains unclear, early recognition of patients with KIU is crucial in successful treatment. Here, we highlight that in severe cases of hydronephrosis particularly in young patients, percutaneous nephrostomy should be used to protect the upper urinary to avoid progression to severe and irreversible urological pathologies. Long-term clinical outcome data is needed in this group of patients in the U.S.
